# Relationships Among Symptoms, Self-Care, and Quality of Life in Individuals With Multimorbidity

**DOI:** 10.1093/geroni/igaf122.3220

**Published:** 2025-12-31

**Authors:** Soo Hyun Kim, Arum Lim, Chitchanok Benjasirisan, Cheryl Himmelfarb, Patricia Davidson, Rebecca Wright, Binu Koirala

**Affiliations:** Johns Hopkins University, Baltimore, Maryland, United States; Johns Hopkins University School of Nursing, Baltimore, Maryland, United States; Johns Hopkins University, Baltimore, Maryland, United States; Johns Hopkins University, Baltimore, Maryland, United States; University of New South Wales Sydney, Kensington, New South Wales, Australia; Johns Hopkins University, Baltimore, Maryland, United States; Johns Hopkins University, Baltimore, Maryland, United States

## Abstract

Multimorbidity is a major concern for aging individuals with heart failure (HF), increasing hospitalization, mortality, and healthcare costs. However, relationships among symptoms, self-care, and quality of life (QoL) remain underexplored. This study analyzed baseline survey data from a cohort study (2022–2023) at a university-affiliated hospital. Participants were aged ≥50 years with HF and at least one additional chronic condition. Socio-demographic and clinical data were collected; symptoms were assessed using the Edmonton Symptom Assessment Scale, QoL via the EuroQoL-5D-5L, and self-care using the Self-care of Heart Failure Index. A cross-sectional regression analysis was performed on a sample of 353 participants, with a mean (±SD) age of 70 (±9.5) years. Among them, 277 (78.5%) identified themselves as their primary caregiver. In the unadjusted analysis, all nine symptoms, self-care maintenance, and self-care confidence were significantly associated with QoL. In the adjusted model, after controlling for age, gender, social support and including all symptoms and self-care domains, pain (β = -0.035, p<.001), shortness of breath (β = -0.011, p<.05), well-being (β = -0.018, p <.01), self-care maintenance (β = 0.004, p<.001), and self-care monitoring (β = -0.001, p<.05) remained significant for QoL. These findings highlight the negative association of symptoms on QoL in those with multimorbidity, while self-care maintenance has positive association with QoL. As multimorbidity becomes increasingly prevalent with aging, further research is needed to better understand the complex relationships among symptoms, self-care, and QoL to inform the development and implementation of effective, tailored multimorbidity management strategies.

